# Dr. Kenneth Wm. Millican

**Published:** 1916-01

**Authors:** 


**Dr. Kenneth Wm. Millican,** B. A., M. R. C. S., L. R. C. P. editor of the London Lancet, died suddenly of acute dilatation of the heart, in London, early in December, aged 62. He was born in Leicester, received his arts degree at Cambridge and studied medicine at St. Mary's Hospital. He was appointed surgeon and laryngologist to the Infirmary for Consumption, London, in 1897 and surgeon to the West End Hospital for Paralysis, London, in 1898. In 1898, he was surgeon to a copper mine in California, having previously traveled around the world. Subsequently, he became one of the editors of the N. Y. Medical Journal. Later he assumed the editorship of the St. Louis Medical Review, resigning in 1906 when this journal changed from a weekly to a monthly and joining the staff of the Journal of the A. M. A. with which he remained till 1910. It was his intention to establish a sanitarium on an estate which he had purchased in Vermont but he was offered a position on the Lancet, which he accepted and held till his death. In addition to his medical writings, he had published two volumes of poetry, Passion Spray and Smoke Clouds, and had frequently favored his friends with original verses.

